# Another Story of O: On the Origin of the Ovule

**DOI:** 10.3390/plants15203112

**Published:** 2026-10-11

**Authors:** Günter Theißen, Robin Schulz

**Affiliations:** Genetics I, Faculty of Biological Sciences, Friedrich Schiller University Jena, 07743 Jena, Germany; robin.schulz@uni-jena.de

**Keywords:** evo-devo, evolutionary novelty, fern, homeobox, innovation, MADS-box, seed

## Abstract

Explaining the mechanisms by which fundamental novelties originated in the history of life is a key goal of evolutionary developmental biology (evo-devo). Several attractive systems for study are provided by land plants (embryophytes), a monophyletic lineage that generated numerous novel reproductive structures within the last 450 million years, such as archegonia, carpels, stamens, and flowers. An especially intriguing case in point is the ovule, yielding a seed after fertilization and thus leading to the seed habit. Ovules and seeds have significantly contributed to the evolutionary “success” of the seed plants (spermatophytes) in terms of species number, biomass, and land coverage. The evolutionary origin of the ovule remains poorly understood, however. One reason is the huge morphological gap between the ovule and the reproductive structures of the closest extant relatives of seed plants, monilophytes (including ferns). So far, the fossil record does not provide an unequivocal and detailed scenario of ovule origin. Here we review what is currently known about the origin of ovules from both paleobotanical and neobotanical points of view. We outline how novel approaches such as phylogenomics and comparative transcriptomics of extant seed plants and ferns currently help to answer a long-standing question: how did the ovule originate?

## 1. Introduction

Ovules are the developmental precursors of seeds. The evolutionary origin of ovules, and hence seeds, has strongly enhanced the reproduction and dispersal of seed plants (spermatophytes), the clade of land plants that produce ovules and seeds. Extant seed plants include extant angiosperms (flowering plants) and gymnosperms. In terms of species number (roughly 300,000 extant angiosperms, about 1000 extant gymnosperms), biomass, land coverage, and ecological relevance, seed plants are by far the most important group of land plants (embryophytes) [1,2]. It is reasonable to assume, therefore, that the origin of the ovule contributed significantly to the evolutionary and ecological importance of seed plants. The importance of seeds and cereal grains (often fruits mainly composed of seeds) for human nutrition and agronomy also cannot be overestimated [3]. Ovules are rather complex structures that encompass several tissues. Defining what an ovule actually is from a morphological point of view is more difficult than it may initially seem. The issue becomes even more complex when questions of homology of certain structures are raised. Since homology is based on common ancestry, answering these questions requires statements about the genealogy and origin of the ovule and its parts. The origin and diversification of something as important as ovules is unquestionable of scientific interest. A number of seminal reviews covering the evolutionary diversification of ovules and seeds in extant and extinct seed plants are already available e.g., [1,4,5]. Here we focus on the crucial question of the origin of the ovule as an evolutionary novelty.

## 2. Evolutionary Novelties

The most impressive feature of evolution is its ability to produce complex novelties, such as eyes and brains (in some animals), flowers and fruits (in some plants). Against the background of physical and chemical laws that may appear somewhat between complicated (classical physics and general relativity), counterintuitive (quantum physics), and seemingly mundane (you name it …), organisms such as roses, mosses, tigers, sheep, chimps and humans originated. How impressive and remarkable the emergence of complex organisms such as animals and plants and their constituent tissues and organs is, probably no one put it better than Charles Darwin more than 160 years ago. “… whilst this planet has gone cycling on according to the fixed law of gravity, from so simple a beginning endless forms most beautiful and most wonderful have been, and are being, evolved” [6].

The concept of evolutionary novelty has been controversially discussed for quite a while, and it has no strict definition that could be considered widely accepted in the scientific community [7,8,9,10]. Some definitions focus on the origin of novel functional capabilities such as vision or flight, whereas others emphasize the origin of novel body parts such as tissues or organs [8]. The distinction between novelty and some other biological concepts, such as apomorphy, a key trait of cladistics, is also not clear. An apomorphy is a derived trait, and may be either a novel character or a novel character state that has evolved from a plesiomorphy (an ancestral form). Since many changes in character state could be simple modifications of an existing character, apomorphy might therefore be a broader term than novelty.

Similarly, one may also ask whether novelty and innovation should be considered as different concepts in evolutionary biology. It has been argued, for example, that the concept of novelty as the developmental genetic mechanisms that bring about new characters, and innovation as the evolutionary process that leads to an ecological and evolutionary success of a clade, should be distinguished [9]. Whether this distinction has heuristic value remains to be determined—in many cases a clear-cut separation may be impossible. It is also questionable to associate novelty and innovation to mechanisms and processes rather than functions and structures.

The impact of novelties on the evolution of organismic lineages is also not fully understood. One might assume that a novelty provides an organismic lineage with advantageous features—vision, flight, and so forth. However, there is often a time lag between the emergence of a morphological or physiological novelty and its subsequent spread and mutational diversification. This suggests that novelty does not necessarily lead immediately to diversification or adaptive radiation [9,11].

Even though it is not entirely clear which kind of evolutionary changes qualify as evolutionary novelties, there is little doubt that novelty, innovation and apomorphy represent interesting phenomena in evolution, and that their underlying genetic and developmental mechanisms deserve explanations. A novelty, by any other name, would remain as challenging to explain.

The difficulty of explaining fundamental novelties, like the different eyes of animals, already concerned Darwin [6]. He argued that complex and unique structures evolve through “an almost infinite number of generations,” during which natural selection gradually fine-tunes these traits. Over long periods, such changes accumulate and bring about the considerable differences that are typical for higher taxa. Darwin’s extreme view is termed here as “strict gradualism”.

How the origin of complex structures could be reconciled with strict gradualism was not obvious to Darwin. He feared that “if it could be demonstrated that any complex organ existed which could not possibly have been formed by numerous, successive, slight modifications, my theory would absolutely break down.” He added, however: “But I can find no such case” [6]. Since then, strict gradualism represents a central tenet of mainstream Darwinism and derived concepts, such as “neo-Darwinism” and the “synthetic theory of evolution” [12,13,14]. However, in contrast to Darwin’s view, strict gradualism is not convincingly supported by empirical evidence. For example, with its often abrupt transitions, the fossil record provides little evidence for a gradual evolution of novelties [14,15]. Also, the topology of phylogenetic trees of both animals and plants does not support a gradualistic origin of numerous novelties [16]. Strict gradualism especially seems to fail where it requires that complex structures originate on a path that implies many intermediate steps that may hamper organisms’ fitness: “What use is half a jaw?”, as Stephen Jay Gould put it [17].

Not surprisingly, therefore, alternatives to strict gradualism have been considered several times since Darwin’s publications. Among them are “mutationism” and different versions of “saltationism” [12,13]. Advocates of these approaches usually do not completely deny gradual change altogether; especially during microevolution and adaptation, they are probably the most frequent, if not the only, mode of evolutionary change. However, they consider them insufficient to explain the origin of fundamental novelties, or species, and higher order taxa [12,13].

Saltationism refers to saltational evolution, which describes profound evolutionary changes occurring within a single generation, or within only a few generations, of organisms. Saltational evolution has been discussed for decades [12,13,17,18,19,20,21], but its relevance remains controversial, and its underlying mechanisms remain largely unknown.

A few decades ago evolutionary biology joined forces with developmental genetics and constituted a new scientific discipline, widely known as “evolutionary developmental biology” (“evo-devo” for short), to better understand the origin of evolutionary novelties. However, “evolutionary developmental genetics” would arguably be a better term because it is actually genes that link development and evolution [22]. According to the evo-devo rationale, morphological novelties in evolution usually result from changes in developmental control genes [18,22,23,24,25], making the inference and study of changes in such genes a high priority in evo-devo studies.

The rapid origin of flowering plants (angiosperms), which apparently left no traces in the fossil record, was considered an “abominable mystery” by Charles Darwin in a letter to his friend Hooker because it was difficult to reconcile with his strict gradualism [26]. Here, we argue that the origin of the ovule of seed plants is another “abominable mystery”—from some perspectives it appears even more abominable than the origin of the flower of the angiosperms.

## 3. Ovules as Evolutionary Novelties

We consider ovules to be more fundamental novelties than flowers. That is why: the flowers of angiosperms represent a syndrome of features, including several novel types of floral organs, such as sepals, petals, stamens and carpels, in special arrangements. Novelty, however, is a relative feature, and nothing in evolution is generated entirely from scratch, but always from pre-existing structures and traits of previous generations. We now know that during the origin of the angiosperms massive recruitment of gene regulatory networks (GRNs) controlling reproductive organ identity occurred, involving several orthologues of floral organ identity, or homeotic, genes [27,28]. According to the “ABCDE” model, homeotic genes determine floral organ identity in a combinatorial way, with class A + class E genes specifying sepal identity, class A + class B + class E genes specifying petal identity, class B + class C + class E genes specifying stamen identity, class C + class E genes specifying carpel identity, and class C + class D + class E genes specifying ovule identity [27]. Remarkably, all known class B genes are members of the clade of DEF/GLO-like genes (also known as AP3/PI-like genes, and often simply termed “B genes”). Likewise, all known class C genes (“C genes”) and class D genes (“D genes”) belong to the clade of AGAMOUS-like (AG-like) genes, all class E genes (“E genes”) are SEP-like genes, and some (but not all) class A genes (“A genes”) are SQUA-like genes (also known as AP1-like genes) [27]. With the exception of some class A genes, all class A, B, C, D and E genes encode MIKC^C^-type MADS-domain transcription factors. These proteins recognize the numerous target genes that they activate or repress to bring about organ identity as dimeric or tetrameric protein complexes (“floral quartets”) [27].

**Figure 1:**
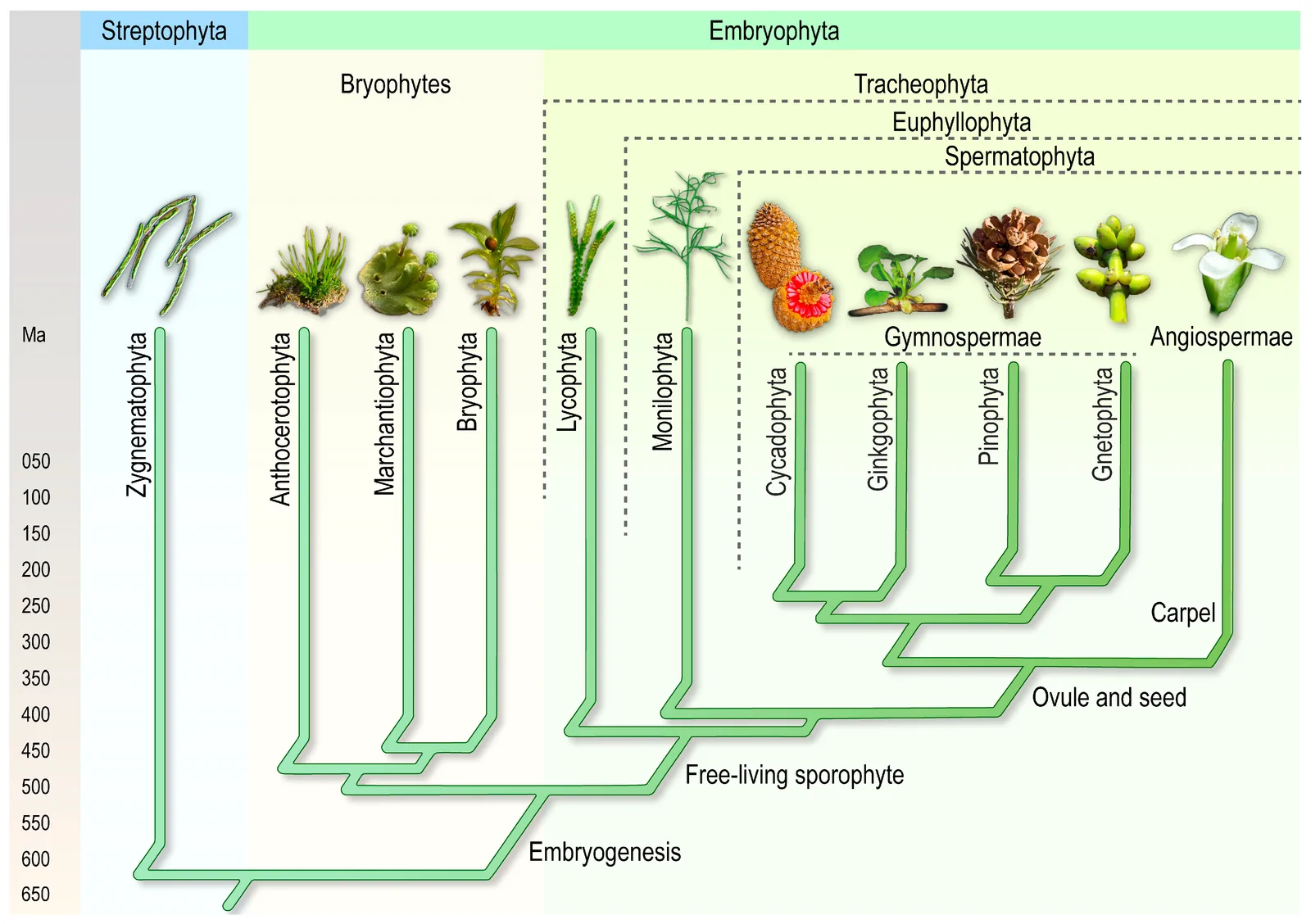
Phylogeny of major lineages of extant land plants (Embryophyta). Phylogenetic relationships (tree topology) and ages of the different clades are as suggested by recent literature [35] except for the relationship of Cycads and Ginkgo, for which several (but not all) recent studies suggest, as shown here, that they represent a clade [2,31,36] and references cited therein. Some evolutionary innovations relevant in this article are indicated at internal branches. The history of land plants began roughly 650 to 600 Ma with plants that gained a haploid–diploid life-cycle and the first multicellular sporophyte. The development of a free-living sporophyte, which became the dominant part of the life cycle, characterized the emergence of the Tracheophyta as a sister clade to the bryophytes. The seed and ovule as new innovations traces back 400 to 360 Ma in the lineage that led to the Spermatophyta. This means that the extant Monilophyta, including ferns and their allies, are the closest extant relatives to all extant seed plants.

Extant gymnosperms are a diverse clade of plants comprising Gnetophyta (Gnetales), Pinophyta (Coniferales), Ginkgophyta (Ginkgo) and Cycadophyta (Cycadales) that, according to molecular data, represent the sister group of angiosperms (Figure 1). All extant gymnosperms have close relatives of class A, B, C, D and E genes (ABCDE genes, for short). Phylogenetic reconstructions suggest that the specific clades to which these genes belong originated in the stem group of extant seed plants about 300–400 million years ago (Ma) [29]. Although informative mutants have not yet been investigated, the spatial and temporal expression patterns of these genes strongly suggest that they have functions in gymnosperms that are very similar to that of angiosperms. Specifically, the AG-like genes (C genes) of gymnosperms are typically only expressed in male and female reproductive organs (in angiosperms in stamens and carpels), but not in vegetative organs, whereas DEF/GLO-like gene (B gene) expression is restricted to male reproductive organs (in angiosperms in stamens) [27]. These findings strongly suggest that reproductive organ identity is very likely controlled in gymnosperm reproductive cones in quite a similar way as in angiosperm flowers: C gene expression defines reproductive organ identity, and B gene expression distinguishes between male (expression on) and female (expression off) organ identity. Even though in some gene clades gene duplications followed by subtle aspects of sub- and neofunctionalization have certainly modified the functions of reproductive organ identity genes, the basic system of reproductive organ identity specification had already been established in the stem group of extant seed plants [27]. Thus, floral organs are derivatives from gymnosperm reproductive organs down to the way in which their identity is specified by developmental control genes. Therefore, the origin of the flower and floral organs, including carpels—the hallmark of the angiosperms—is not as mysterious anymore as it may have appeared to Charles Darwin, although many details still need to be clarified [28,29,30].

Concerning an understanding of their origin the situation is considerably different for ovules and the seed habit compared to flowers and their individual organs, however, because close relatives of ovule-identity genes have not yet been identified in non-seed plants [29,31]. The sister group to extant seed plants represents the Monilophyta (monilophytes), including ferns and horsetails (Figure 1). Recent analyses have revealed that fern genomes contain roughly as many MIKC^C^-type MADS-box genes as seed plants do, e.g., [32,33], but none appears to fall into the 12 seed-plant-specific clades of MIKC^C^-type MADS-box genes previously identified [29], including the ones containing the class A, B, C, D and E genes. So, there may not have been floral organ identity genes, including class C, D and E genes specifying ovule identity, in the most recent common ancestor (MRCA) of monilophytes and seed plants that could have been directly recruited for seed origin. ABCDE genes first had to originate from other MIKC^C^-type genes by gene duplication and divergence in sequence, expression and function in the stem group of extant seed plants before they could function as the reproductive organ identity genes known today. How exactly this happened could not be determined until now due to the low resolution of the deep branching of the reconstructed phylogenetic trees of MIKC^C^-type genes.

According to recent analyses there have very likely been close relatives of genes present in the MRCA of monilophytes and spermatophytes that have an important role in seed development and hence possibly also seed origin and evolution. Examples are *DELAY OF GERMINATION1* (*DOG1*) and *LEAFY COTYLEDON1* (*LEC1*) that have been identified in mosses and ferns, respectively [34]. However, none of these genes appears to specify ovule identity and hence would qualify as ovule identity genes.

A large morphological gap divides the monilophytes from the seed plants, especially with respect to reproductive organs. There is nothing in monilophytes that can reasonably be considered homologous or homonomous to the spermatophyte ovule as a whole, the homology of some parts, such as the megaspore mother cell or the megaspore notwithstanding. We argue, therefore, that the ovule did not originate just by modification of pre-existing organs, but represents an evolutionary novelty in a strict and fundamental sense, thus meeting some but not all contemporary definitions of “evolutionary novelty” [7,8,9,10,11].

Given the apparent absence of C, D and E genes in the MRCA of monilophytes and spermatophytes but the presence of ABCDE genes in the MRCA of spermatophytes, the origin of the seed may thus have required more developmental genetic innovation than flower origin, at least with respect to organ identity specification. Therefore, the origin of the ovule may appear even more mysterious than the origin of the flower. In any case, the origin of the ovule and seed has not only been one of the most significant events in the history of land plants but is also one of the remaining “black-boxes” in our understanding of land plant evolution.

## 4. The Structure and Development of Ovules

What actually is an ovule? There are surprisingly different (e.g., structural, developmental, and phylogenetic) ways to define it, even though they obviously refer to the same “thing”—or at least they should. For an initial orientation, we start with simple definitions: an ovule is an indehiscent integumented megasporangium; a fertilized ovule is a seed [2].

In any case, an ovule is a complex structure that encompasses several tissues with different functions and ploidy levels depending on the developmental stage.

**Figure 2:**
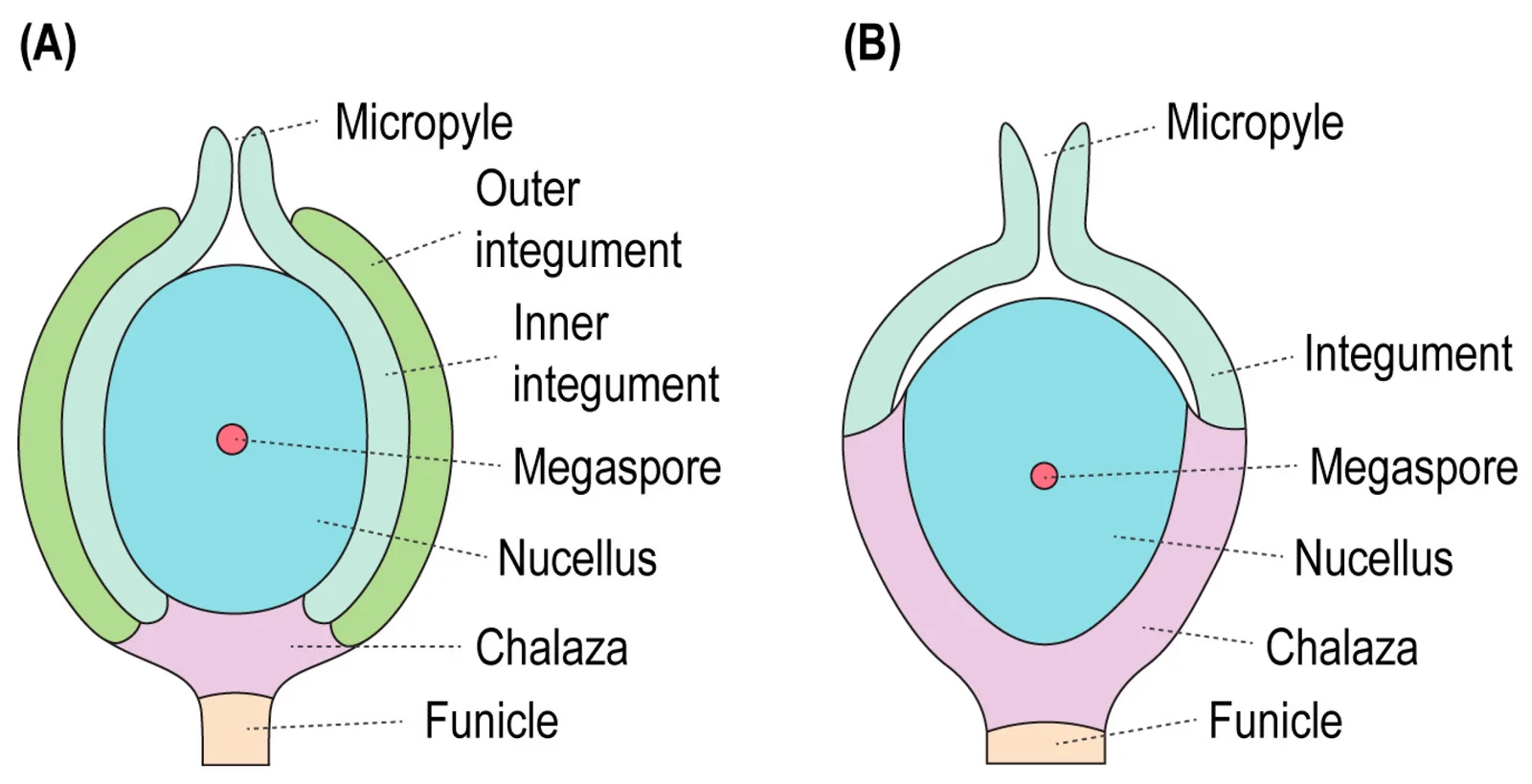
Generalized structures of a bitegmic orthotropus (or atropous) angiosperm ovule (**A**) and a gymnosperm ovule (**B**). Sketches colored and labelled to show the different structures and tissues. Figure according to [5,40], modified.

The structural “core” of the ovule is the nucellus, which represents the megasporangium of the seed plants (Figure 2) [1,5,37]. The nucellus is enveloped by one (in case of gymnosperms) or mostly two (angiosperms) covering layers named integuments (Figure 2) [1,5], which are not necessarily homologous structures when different major lineages (ginkgo, conifers, cycads) are compared [38].

Ovule development starts from maternal tissue—in case of angiosperms the placenta within the carpels of the gynoecium—with the initiation of the ovule primordium. After multiple anticlinal and periclinal cell divisions the primordium differentiates into three regions, funicle (funiculus, stalk), chalaza (base of the ovule) and nucellus (Figure 2). The funicle connects the ovule with the placenta or other maternal tissue and eventually provides nutrients to the gametophyte once it has developed. The chalaza initiates the integument(s), (a) laminal structure(s) essential for the formation of the nucellus by enveloping it [37]. While most angiosperms have an outer and an inner integument, gymnosperms have only a single integument (considerable diversity between the integuments of different gymnosperm lineages notwithstanding). The inner or single integument forms an ancestral tubular sheath around the nucellus, while the outer integument in angiosperms is a later evolutionary innovation [5].

In the process of sexual reproduction in angiosperms, a pollen tube grows through the ovary and comes out at the placenta. For fertilization, the pollen tube must deliver sperm into the embryo sac, using one of three main pathways. In most extant angiosperms, the pollen tube enters directly through the micropyle, a structural opening at the apex of the integuments; this process is termed porogamy and might represent a derived state of pollen tube entry. In case of chalazogamy, the pollen tube bypasses the micropyle and enters through the chalaza at the base of the ovule and hence opposite to the micropyle. In yet another scenario, termed misogamy, the pollen tube pierces itself through the integuments or the funiculus. After being guided by signal molecules in its growth the pollen tube releases a sperm cell to fertilize the egg cell [39]. After fertilization the integuments develop into the seed coat, which protects the embryo [37].

The ovule is initially composed exclusively of diploid, sporophytic, maternal tissue, including a megaspore mother cell which develops within the nucellus. The megaspore mother cell generates four haploid cells by meiosis, three of which typically degenerate soon. In some cases of bisporic and tetrasporic female gametophytes, however, such as in *Gnetum*, megaspores do not degenerate.

The remaining one represents the megaspores which develop into megagametophytes (female gametophytes) by mitotic cell division. The mature megagametophyte of gymnosperms is multicellular, and several archegonia typically develop within it, each of which produce one egg cell [1]. Again, as so often in biology, there are exception, however, such as in *Araucaria*, in which only one archegonium develops in a megagametophyte.

In most angiosperms, the mature female gametophyte, usually called an “embryo sac”, comprises just seven cells and eight nuclei, representing the seven-celled and eight-nucleate Polygonum-type. In some early-branching groups of angiosperms, however, the embryo sac is four-celled and four-nucleate (Nuphar/Schisandra-type), which is thought to be the ancestral type [39]. One of the seven (or four) cells of the embryo sac is the egg cell; the others are two synergids, three antipodal cells, which are absent in the Nuphar/Schisandra-type, and one central cell. The fertilized egg cell develops into an embryo. In angiosperms, a second sperm cell fertilizes a diploid central cell nucleus of the embryo sac, giving rise to the development of a triploid (secondary) endosperm. In case of four-celled embryo sacs, the endosperm is only diploid, however. In case of gymnosperms the endosperm develops directly from the megagametophyte and is hence termed the primary endosperm.

A mature seed is equipped with a drought-resistant embryo, an often nutrient-rich endosperm that nourishes the embryo during early germination, and a protective seed coat. Several mechanisms control seed dormancy, ensuring that germination occurs only under favorable conditions [1]. These remarkable features of seeds have plausibly contributed to the ecological and evolutionary dominance of the spermatophytes across most terrestrial habitats.

The ovule is often considered the female reproductive structure of the seed plants. However, since the egg cell develops in the female gametophyte, it appears more appropriate to regard the embryo sac as the female reproductive structure *sensu stricto*, notwithstanding the fact that it forms part of the ovule. A similar argument could be made for carpels as female reproductive structures, with carpels including ovules including female gametophytes harboring egg cells.

## 5. Paleobotany: What Does the Fossil Record Tell Us About the Origin of the Ovule?

Seeds, and hence also their developmental precursors, ovules, are the defining feature of the spermatophytes (seed plants), namely angiosperms and gymnosperms (Figure 1). In angiosperms, ovules are considered integral parts of the carpel—“angiosperm” means “vessel seed”, referring to the fact that the ovule and hence the seed develops within the ovary, to which one or several carpels contribute. By contrast, gymnosperms develop “naked seeds” on scales or modified leaves, often organized into cones or strobili. Since ovules and seeds characterize seed plants and are present in all gymnosperms, including stem group gymnosperms, they are evolutionarily more ancient than angiosperms. Ovules are also specified by a unique combination of organ identity genes [27] and could therefore be considered as organs in their own right. In any case the development of ovules and seeds clearly separates the spermatophytes from all the non-seed plants, including monilophytes, lycophytes, and bryophytes.

Several lines of paleobotanical as well as molecular evidence suggest that seed plants (spermatophytes) are a monophyletic group (clade) within the euphyllophytes, a group of vascular plants that is characterized by the possession of “true leaves” (“megaphylls”) [31,41]. Seed plants originated very likely within the Devonian (about 419–359 Ma) and thus surprisingly early in the evolution of land plants; the exact age, however, is not known. According to different authors, seed plants can be traced back to the Famennian (372–359 Ma) of the Late Devonian, to the Givetian (388–383 Ma) of the Middle Devonian, or even to the Pragian (411–407 Ma) of the Early Devonian [1,41,42,43] and references cited therein. The closest relatives of the seed plants among seed-free plants are hence probably all extinct Devonian plants. Intermediate stages documenting the origin of ovules are not available in extant plants—extant non-seed plants lack ovules, whereas all extant seed plants develop fully evolved ovules. One key to understanding ovule origin therefore lies in the fossil record, which is notoriously incomplete and difficult to interpret, however; needless to say, this hampers our understanding of euphyllophyte evolution. Two groups of extinct euphyllophytes have been discussed as precursors of seed plants, progymnosperms and Stenokoleales [41]. Progymnosperms were trees with a free-sporing, fern-like mode of reproduction, but gymnosperm-like (pycnoxylic) wood. Stenokoleales are characterized by actinosteles with protoxylem parenchyma and by axes bearing appendages bifurcated at the base. Among progymnosperms, aneurophytes have been considered more likely to contain the ancestor of seed plants than the second group, archaeopterids. The reason is that the earliest genuine seed plants known, such as *Elkinsia*, possessed actinosteles, like aneurophytes, and not complex eustele-like or polystelic systems like the archaeopterids, or eusteles proper like younger spermatophytes, respectively. Stenokoleales also had actinosteles [41].

Parsimony analyses of morphoanatomical characters applied to an extensive taxon sampling of fossil plants recovered Stenokoleales and aneurophytes as clades; aneurophytes were placed as sister to a “bilateral clade” that includes Stenokoleales, *Actinoxylon banksii*, monophyletic seed plants, and some other taxa [41]. Thus, seed plants might be more closely related to (at least some) Stenokoleales than to aneurophyte progymnosperms (whether Stenokoleales represent a clade is controversial in the literature). Concerning ovule origin, there is one caveat, however. Among the nine seed plant species included in the comprehensive study by Toledo et al. [41], only one (*Elkinsia polymorpha*) is identified as having attached reproductive structures, i.e., ovules or seeds (the difference is often not recognizable in the fossil record)—the other eight are just assumed to be seed plants because of similarities in vegetative characters. So, there is only circumstantial evidence, at best, that they are really seed plants in a literal sense.

Given its complexity, it appears likely that the spermatophyte ovule originated in several steps. According to a long-held hypothesis, the first step involved the transition from homospory, meaning the production of just one kind of spores of equal size, to heterospory, the production of two types of spores, small microspores and larger megaspores, formed in two morphologically divergent sporangia.

Each megaspore mother cell within a megasporangium produces four equal megaspores by meiosis. Further independent evolutionary changes may have led to the elimination of three of them and retention of only a single megaspore in the megasporangium (monomegaspory), probably to avoid competition for space and nutrients among four female gametophytes.

The single megaspore was not dispersed, but was retained within the megasporangium, where it eventually develops into a megagametophyte containing an egg cell (endospory, i.e., gametophyte development within the spore wall).

**Figure 3:**
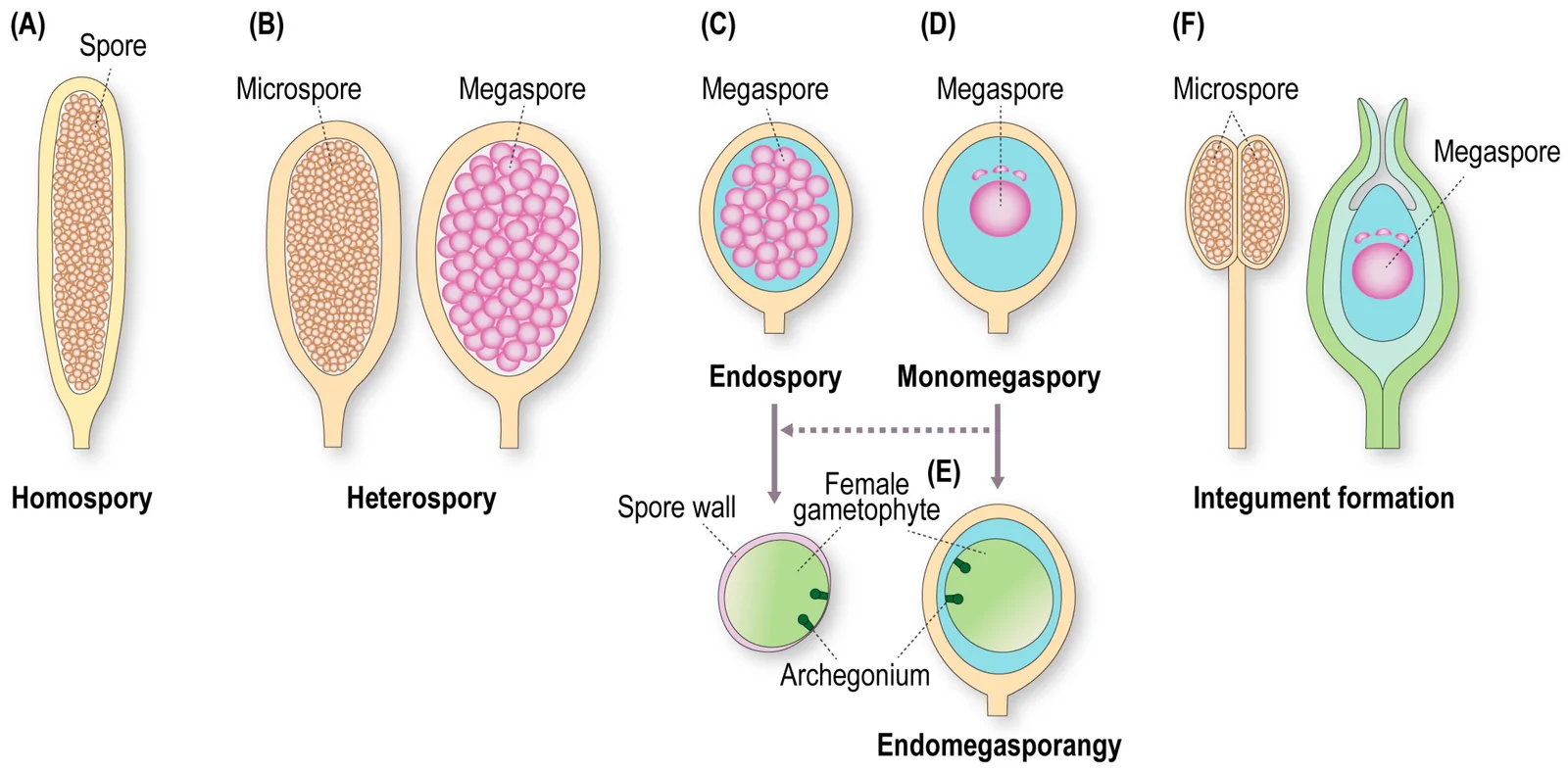
Potential innovations involved in the evolution of the ovule from a homosporous ancestor. Ovule evolution involved the acquisition of several distinct reproductive characters, although these characters did not necessarily arise in a single invariant sequence. (**A**) Homospory represents the ancestral condition from which heterosporous vascular plant lineages evolved. (**B**) Heterospory is the production of two distinct spore types, microspores and megaspores, which develop into male and female gametophytes. Heterospory occurs in some lycophytes and monilophytes and in all seed plants. (**C**) Endospory is the development of the gametophyte within the spore wall. (**D**) Monomegaspory is the production of a single functional megaspore within each megasporangium, typically through the abortion of the other products of meiosis. (**E**) Endomegasporangy is the retention of the single functional megaspore within the megasporangium instead of its release as a free spore. (**F**) Integument formation enclosed the megasporangium within one or more layers of sterile tissue while leaving an apical opening, the micropyle. The diagram presents a conceptual reconstruction of character acquisition rather than a confirmed universal evolutionary sequence [2,5,44].

Finally, integuments surrounding the megasporangium originated, possibly by fusion of terminal branchlets of a dichotomously branched axis, termed telomes (megasporangium integumentation). This way tightly locked, the megasporangium may have become fully indehiscent, and the ovule was established, providing a better protection for the egg in terrestrial environments, and enabling the development of seeds. Or so the story goes (Figure 3) [1,4,44,45,46].

Even though this scenario appears plausible, it is still highly speculative because relevant intermediate states that could be unequivocally assigned to the lineage leading to extant seed plants are unknown from the fossil record. It has also been argued that heterospory and gametophytic unisexuality are not necessarily antecedents of endospory but may have arisen as developmental consequences of endospory [47].

Among the first plants that developed ovules in evolution were the extinct “seed ferns” (pteridosperms) (extant cycads have been included in pteridosperms in some analyses) [48]. Note that the term “seed ferns” and “pteridopserms” are misleading since these plants are gymnosperms rather than ferns. Pteridosperms are probably paraphyletic and have been considered the “backbone of seed plant phylogeny”, even though their phylogeny is not well resolved. This highlights the importance of extinct taxa known only from fossils for understanding seed plant phylogeny [41,48].

It has been pointed out that the earliest, most ancient ovulate structures found in the fossil record should be better considered as “preovules” rather than ovules proper, because their nucellus is surrounded by unfused or only partially fused integumentary lobes; these structures hence lack a micropyle proper. Preovules may have provided micropyle-like functions by modifications of the nucellar apex [49].

One of the earliest representatives of taxa producing preovules might have been *Runcaria heinzelinii* [43,49]. According to some scientists its preovules consisted of a short basal cup-like cupule composed of four segments. Based on this view the cupule contained a radially symmetrical megasporangium surrounded by an integument of 16 or more free lobes. The megasporangium bore a long distal extension emerging above the lobes and enlarged at the top. This structure probably played a role in catching microspores. It seems reasonable, therefore, that *Runcaria* reproduction involved anemophilous pollination and dissolution of sporangial cells to enable fertilization. This combination of physiology and morphology is quite unique with respect to later appearing ovules and seeds [43,49]. Note that according to other researchers, however, the presence of a single functional megaspore, its indehiscent status within the sporangium, and even the status of a source plant as a vascular land plant is questionable [2]. This case exemplarily demonstrates that lack of data often seriously hampers the interpretation of fossils, even in case of iconic plants such as *Runcaria*.

In the Late Devonian preovules were already quite diverse. Some (according to one view all [49]) may have developed the hydrosperman syndrome, a set of modifications at the nucellar apex. In this case the nucellar apex is modified as a pollen chamber closed by a plinth and extended by a cylindrical structure termed salpinx. The pollen chamber contains a central parenchymatous column. The whole modification of the nucellar apex has been termed “lagenostome” [49].

The different preovules of the Late Devonian show different degrees of fusion of the integumentary lobes or characteristics of the cupule (vegetative segments that typically enclose the preovules).

The Late Devonian ovules have been classified into different types. One system of classification was presented more than two decades ago by Prestianni [49]. The Moresnetia-type of preovules is characterized by a four-unit cupule generated by two successive cruciated dichotomous divisions. A progressive fusion of the integumentary lobes can be observed, ranging from completely dissected (as in *Moresnetia zalessky*) via an intermediate degree (*Xenotheca devonica*, *Elkinsia polymorpha*) to an almost completely fused integument (*Archaeosperma arnoldii*). Whether this apparent series of fusion events represents a genuine evolutionary trend has remained unknown for several reasons, however [2,49].

Two other types of preovules are the Warsteinia- and Dorinnotheca-types [49] and references cited therein. *Warsteinia paprothii* is acupulate; its integument is made of four winged lobes fused or adnate to the nucellus. *Dorinnotheca streelii* developed cupules composed of eight parts fused proximally so that they form a cup, the segments of which divide to form at least 40 free endings. Each cupule contains only one central ovule. The integument is composed of four free terete lobes. Both Warsteinia-type and the Dorinnotheca-type preovules might have been anemochoreous [49].

A fourth type of preovules is the Condrusia-type, based on species such as *Condrusia rumex* and *C. minor* [49] and literature cited therein. *Condrusia* ovules differ from all the others in the characteristics of their cupule. The *Condrusia* cupule is composed of two flat bilaterally symmetrical wings adpressed against each other. In the two species mentioned the integument is not dissected into lobes and shows a complex organization of three layers, with the middle one consisting of large sclerified cells. The nucellus appears to be fused with the integument. Its apex is modified into a very long salpinx protruding above both the integument and the cupule wings [49].

Another type of preovule, the acupulate Aglosperma-type, based on *Aglosperma quadrapartita* and *A. avonensis,* had integuments formed by three to four flat lobes fused up to their lower third [49]. Note, however, that meanwhile *Aglosperma* has been put into other genera and species.

Some more types of fossil preovules and seeds cannot be easily put into any of the mentioned categories [49]. The age and order of appearance of the different types of preovules mentioned is not fully clear, even thought it has been maintained that the Morestian-type is the oldest one [49].

For quite some time known fossils of ovules mainly traced back to the ancient Laurussian continent (that later gave rise to North America and the main part of Europe), leading to the impression that the earliest seed plants had small (generally less than 5 mm long) (pre)ovules that were born in a cupule (possibly involved in protection and pollination); these cupules were either uni- or multi-ovulate [46]. Surrounding and overtopping several aggregated ovules, multiovulate cupules may have prevented the ovules from losing too much water in dry conditions.

However, numerous fossils of (pre)ovules more recently reported from a lagerstätte in South China have significantly modified and widened our view about ovule diversity in the Late Devonian; for a comprehensive review, see [42]. For example, in contrast to the cupulate, often multiovulate cupules of Laurussia, many spermatophytes on the South China plate developed ovules without a cupule, with integumentary wings and plumes possibly functioning in wind dispersal or photosynthesis (rarely known among Laurussian ovules) [42] and references cited therein. The (pre)ovules of what later became South China were also often considerably larger (up to 1 cm) than those of Laurussia.

The differences between typical Laurussian and South China block (pre)ovules probably reflect climatic differences between the different growth habitats. Several lines of circumstantial evidence suggest that Famennian seed plants, at least those on the South China plate, mainly inhabited dry inland settings such as uplands rather than wet lowlands and coastal habitats where, e.g., lycopsid forests had thrived [42].

However, both the Laurussian continent and the South China block were located in lower latitudes and/or near the equator, and in an arid and tropical climate belt, respectively [42].

All in all, the fossil record suggests that Famennian seed plants developed terminally-bore (pre)ovules on terminal branches; these (pre)ovules were somewhat primitive in that they developed a lobed integument without a micropyle and usually had hydrosperman reproduction through a pollen chamber [42] and references cited therein. The seed plants that produced these structures were abundant at genus level (20 genera known from Laurussia, 12 from South China) and widely distributed on the Laurussian continent and the South China plate. However, the floras between Laurussia and South China were quite different in composition and distinct in interesting traits, including size of ovules, possession of ovulate cupules and developing integument functions such as wind dispersal and photosynthetic nutrition [42].

Interestingly, after the Devonian, there is a remarkable gap in the seed record; it is somewhat mysterious, since quite a number of vegetative parts that have been assigned to seed plants were found, but almost no reproductive organs [2,47,48,49]. The reasons for that are largely unknown, but irrelevant for an understanding of the very origin of the ovule(the focus of this article). However, the detailed analysis of the development of some ovules from the Carboniferous (ca. 359–299 Ma) has recently thrown some light on the still enigmatic nature of the cupule. The respective study suggests that cupule, integument and nucellus together developed in a shoot-like fashion, possibly ancestral to the reproductive shoots of extant seed plants [50].

Taken together, the fossil record reveals an impressive diversity of types of preovules and hence the rapid early diversification of the ovule and seed to a certain extent. However, it tells us so far less about the very origin of the ovule, except that a preovule stage probably preceded the origin of the fully evolved ovule. How preovules originated remains unknown, however, since there are no intermediates between progymnosperm sporangia and hydrosperman preovules known from the fossil record [46]. Specifically, the fossil record does not distinguish between several hypotheses of ovule origin based on the evo-devo rationale. Given the delicate structural changes that probably occurred, however, this may be asking too much of the fossil record.

## 6. Evolutionary Developmental Genetics Views on Ovule Origin

According to the rationale of evolutionary developmental biology (evo-devo) the origin of structural evolutionary novelties in multicellular organisms requires changes in developmental processes. Accordingly, several hypotheses have been developed that link (more or less explicitly) development and evolution to explain the origin of the ovule.

An early attempt suggested that terminal branchlets (telomes) of a dichotomously branched axis surrounding the megasporangium fused and thus generated the integuments, a hypothesis heavily based on the telome theory of Walter Zimmermann [2,46] and references cited therein.

Mechanistically, developmental and evolution are linked by the evolution of developmental genes, suggesting that genes that control the development of structures (e.g., tissues and organs) have also been of crucial importance during the evolution of these organs—the rationale of evodevotics [22]. Accordingly, more recent hypotheses have taken genes more or less specifically into account. For example, based on the expression of some genes such as the homeobox gene *WUSCHEL* (*WUS*) it has been hypothesized that the ovule represents its own kind of meristematic axis, and that hence the integuments of the ovule (and sterile appendages of preovules) could be lateral organs initiated by the nucellar meristem; integuments might hence be of *de novo* origin rather than derived from preexisting structures such as sterile (or sterilized) telomes as mentioned above [51].

The meristematic nature of at least part of the ovule has recently been corroborated by transcriptomic analysis. Profiling dynamic gene expression in ovule development of representative species from all four major groups of extant gymnosperms supports the hypothesis that early formed chalaza regions function indeed as a meristematic zone, giving rise to the formation of nucellus and integument in a manner similar to that of the activity of the shoot apical meristem [40].

A large-scale analysis of gene expression in the fern model system *Ceratopteris richardii* was taken as circumstantial evidence that seed developmental genes were co-opted from sporangia developmental networks [32].

Two clades of MIKC^C^-type MADS-box genes, the AGAMOUS- (AG-) like genes and the B_sister_ genes, are of considerable importance in an evodevotics framework of ovule origin. Members of these two clades control essential aspects of ovule and seed development in some flowering plant model systems and, by inference, probably throughout the seed plants.

According to the ABCDE model of floral organ identity, ovule identity is controlled by a combination of class C and D MADS-box genes, both of which are closely related members of the clade of AG-like genes [27,52,53]. The phylogeny of AG-like genes, involving sub- and neo-functionalizations following several gene and genome duplications in gymnosperms and angiosperms, has not been fully resolved. Therefore, the evolution of the C function (specifying stamens and carpels) and of the D function, which specifies ovules, has remained unclear [22,27,54]. In considerations of organ identity such as this, carpels and ovules are assumed to be different organs, whereas, as mentioned above, botanists consider ovules to be genuine parts of carpels in angiosperms. In addition to their basic functions in specifying carpel and ovule identity, some C and D function genes have obtained additional functions during evolution, such as *SHATTERPROOF1* and 2 (*SHP1* and *SHP2*) in fruit dehiscence, and *SEEDSTICK* (*STK*) in seed abscission. These genes are all from of *Arabidopsis thaliana* [55]. *AG*-like genes are absent in non-seed plants [31]. Since they function as ovule identity genes at least in angiosperms, and very likely also in gymnosperms, the origin of these genes might be closely, possibly even causally, linked to the evolutionary origin of ovules and seeds.

The same could apply to B_sister_ genes, another ancient clade of MIKC^C^-type MADS-box genes. Historically, these genes are also known as GGM13-like genes or AGL32-like genes. B_sister_ genes represent the sister lineage of B genes; in contrast to B genes, which are mainly expressed in male reproductive organs and angiosperm petals, B_sister_ genes are almost exclusively expressed in the female reproductive organs and seeds of spermatophytes; both aspects contributed to the naming of these genes [56]. Canonical B_sister_ genes such as *ARABIDOPSIS BSISTER* (*ABS*) of *A. thaliana*, appear to specify the identity of the endothelium, i.e., the innermost layer of the inner integument of ovules [57]. However, the single *abs* mutant phenotype does not reveal the full function of the *ABS* gene, since it is partially masked by redundancy with the D gene *STK*; in *abs stk* double mutants, ovule function is seriously compromised: the endothelium is completely absent and massive starch accumulation occurs in the embryo sac [58]. This redundancy is quite remarkable, since B_sister_ and D genes belong to different clades of MIKC^C^-type genes that separated very likely more than 300 MYA [29]. However, B_sister_ genes have been strongly conserved throughout seed plant evolution—there seems not to be a single seed plant species without a B_sister_ gene, at least none has been studied so far [59]. This is difficult to reconcile with a largely redundant function of B_sister_ genes.

There is evidence that *OsMADS29*, one of the three B_sister_ genes in rice, regulates the degradation of the nucellus during seed development [60,61]. Such a function was later also found in *A. thaliana*, where it may have been hidden by the redundancy of *ABS* and its paralogue *GORDITA* (*GOA*) [59,62]. These B_sister_ genes, and many others, are expressed throughout the ovules of gymnosperms, including the nucelli [63]. Taken together, these data suggest that the elimination of nucellar structures might be a crucial, conserved, and ancestral core function of B_sister_ genes throughout seed plants for about 300 million years. Since the nucellus is the functionally most crucial part of the ovule, an important function in nucellus development is easily conceivable as (one of) the ancestral function(s) of B_sister_ genes.

As mentioned above, the nucellus differs from the megasporangia of non-seed plants such as ferns and their allies in a very remarkable feature, that is megaspore retention. Retention of a megaspore in the indehiscent megasporangium requires at least partial degeneration of the nucellus later in development to leave room for growth of the female gametophyte, the developing embryo, and endosperm [45,64,65,66]. It could therefore well be that B_sister_ genes provided a crucial function during the origin of the ovule, that is the control of nucellus elimination. The origin of the seed habit might thus be tightly linked to the evolution of B_sister_ genes. Therefore, we hypothesize that both C and D genes (which are all AG-like genes) and B_sister_ genes hold a key to a better understanding of some of the most impressive novelties of land plant evolution: seeds.

Beyond MIKC^C^-type MADS-box genes, among the many other gene families involved in ovule development, we want to highlight the subfamilies of *KNOX* and *BELL* genes here, which are both members of the homeobox gene family. *KNOX* and *BELL* genes encode homeodomain proteins of the three-amino-acid-loop-extension (TALE) superfamily. They are especially interesting because of their wide range of interaction partners and their importance for meristem identity and organ development, including that of the ovule [67]. Additionally, they are involved in post-fertilization networks and are key regulators for transitioning between the gametophyte and sporophyte [68]. During evolution, the *KNOX* gene family has split into class I and class II [69]. Homologous representatives of both classes, as well as of the *BELL* gene family, can be found in bryophytes, lycophytes, monilophytes, and seed plants [70]. In fact, homologues of *KNOX* and *BELL* can already be found in chlorophytes like *Chlamydomonas reinhardtii,* where proteins they encode form a heterodimer as part of the post-fertilization network and are essential for zygote development [71].

A loss-of-function study of class I *KNOX* genes in *Arabidopsis thaliana* showed that the mutants could be fully complemented by the expression of genes from bryophytes or lycophytes but only partially complemented by genes from monilophytes, indicating that class I *KNOX* genes might had a broader range of functions in earlier plant lineages. Furthermore, the lineage of *BELL* genes has evolved alongside the *KNOX* genes, acquiring more specialized functions in monilophytes and seed plants [72].

Seed plants have in comparison to non-seed plants a large number of paralogous *BELL* and *KNOX* genes [73]. For instance, in the angiosperm *A*. *thaliana, BEL1* is highly expressed in the ovule where it controls the development of the integuments and it suppresses the expression of *AG*. Consequently, the knockout of *BEL1* caused the transformation of ovule integuments into carpel walls [74]. Among the class I *KNOX* genes, *SHOOT MERISTEMLESS* (*STM*) in *A. thaliana* is expressed to maintain meristem identity by restricting cell differentiation, whether it is the shoot apical meristem or the carpel marginal meristem to generate ovules [51,67]. The role of class II *KNOX* genes in seed plants is still partially unclear, but they seem to act antagonistically to class I *KNOX* genes. By promoting cell differentiation, they may allow for more sophisticated gene regulatory networks and may enable a higher degree of morphological complexity [68].

There is evidence that in bryophytes *BELL* and *KNOX* genes are important for the post-fertilization network and phase transition between the gametophyte and sporophyte. In the moss *Physcomitrium patents* homologous genes of *KNOX2* are needed to maintain the sporophyte. Interestingly, a *KNOX2* knockout mutant caused the development of gametophytic features at the sporophyte stage [75]. Also, *P. patens* has a homologue of *BEL1* which is a master regulator of the transition of gametophyte to sporophyte [73]. In addition, overexpression of *BEL1* in gametophytic cells causes the formation of embryos, resulting in reproductive diploid sporophytes without prior fertilization [73]. In the hornwort *Anthoceros agrestis*, both *KNOX2* and *BELL* genes are expressed in the sporophyte stage; however, *BELL* is expressed during the early stages and *KNOX2* during the later stages, indicating no direct interaction. Interestingly, *KNOX1* orthologues have been lost. The function of the remaining *KNOX* genes in *A. agrestis* is not yet fully understood, but it is believed to be part of the sporophyte identity [76]. In the liverwort *Marchantia polymorpha*, *KNOX* is expressed in the egg cell while *BELL* is expressed in the sperm cell. As in *C. reinhardtii*, the proteins form a heterodimer after fertilization which is needed for zygote development. These findings suggest an ancestral role for *KNOX*/*BELL* interaction in immediate post-fertilization processes, which later in evolution shifted toward meristem maintenance [77].

Regarding lycophytes as the sister group of euphyllophytes, uncertainties remain about the specific functions of *BELL* and *KNOX* genes. A study on *Selaginella moellendorffi* showed that all *KNOX* genes are expressed in stem and leaf tissues of the sporophyte [78], while another study on *Selaginella kraussiana* showed interaction of KNOX proteins with other transcription factors within the shoot apical meristem [79]. In the fern *Ceratopteris richardii*, a member of the monilophytes, which are the closest relatives to seed plants (Figure 1), two class I and one class II *KNOX* genes could be identified. The class I genes are, like in seed plants, expressed in the shoot apical meristem, while the class II gene is expressed in a wider variety of tissues. Although it can be assumed that their function in the sporophyte is similar to those in seed plants, it was shown that meristem identity in the gametophyte of *C. richardii* does not depend on class I *KNOX* genes [80]. On the other hand, a study on the expression patterns of *KNOX* and *BELL* in the fern *Vandenboschia speciosa* revealed that homologues of genes of both *KNOX* classes can be found predominantly expressed in either the gametophyte or the sporophyte, while *BELL* is expressed in both the gametophyte and the sporophyte. The function of *KNOX* and *BELL* in *V. speciosa* could be the maintenance of meristems and as key regulators in post-fertilization for gametophyte–sporophyte transition. Additionally, they could be repressing sexual development and initiating vegetative propagation of the gametophyte [81]. Overall, there are many genes involved in plant sexual reproduction, but *KNOX* and *BELL* are found across all plants and act as important key regulators for meristem identity. It remains to be seen what can be learned from functional studies in lycophytes and, more importantly, in monilophytes, to understand their link to seed plants and how it might connect to the origin of ovules.

Compared to the evolutionary origin of the ovule, which is still quite a mystery, considerably more is known about its developmental origin. According to the evo-devo rationale, gene regulatory networks controlling ovule development may have been recruited or established during the evolutionary origin of the ovule, a principle that goes certainly also beyond organ identity as discussed above.

Many gene lineages directly involved in ovule development, including *BEL1*, *KAN*, *UCN* and *SHORT INTEGUMENTS1* (*SIN1*), evolved as a result of whole-genome duplication events (WGD) along with the radiation of angiosperms. These genetic pathways are conserved in all primary lateral organs but their mode of action is highly flexible depending on spatio-temporal expression patterns [82]. Homologues of these genes have been identified in non-seed plants like the monilophytes and lycophytes [83] likely performing there an ancestral function that originated before the invention of ovules.

That ovules are complex structures does not necessarily mean that their origin required massive genetic changes. Sometimes mutation or recruitment of single genes through minor genetic changes, for example, in promoter regions, or in sequences encoding protein-interaction domains, may initially do. Later, refinements by mutation and selection are certainly required. Similar arguments could be made for the mutation or recruitment of gene regulatory networks.

Based on a detailed review of fossil evidence and *Arabidopsis* mutants, it was recognized that mutations in single homeotic genes, such as *BEL1*, can bring about some seemingly “primitive” (putatively ancestral) features in ovules [4]. For example, in *bel1-3* mutants, the inner integuments do not form [4]. It is therefore tempting to speculate, that characteristic changes in ovule structure may have been based on mutational changes in single homeotic genes.

The loss of megasporangium dehiscence and hence the retention of the megaspore, coupled with partial degeneration of the nucellus to enable female gametophyte growth, have been considered crucial processes during the origin of the ovule [45]. Whereas changes in B_sister_ MIKC^C^-type MADS-box genes might have been important for nucellus degeneration, it seems likely that a different factor has been involved in megasporangium dehiscence [45].

Yet a completely different scenario is outlined by the “golden-trio hypothesis”. Based on studies on the fern *Adiantum capillus-veneris*, it was suggested that the developmental program of the seed arose from the spatiotemporal integration of three physiological and genetic components: assimilate flow, stress responses mediated by the phytohormone abscisic acid (ABA), and stress-induced expression of the gene *LEAFY COTYLEDON1* (*LEC1*) [84].

Given the obvious diversity of concepts it becomes apparent that we are still quite far away from a comprehensive understanding of the developmental and genetic mechanisms that generated ovules in evolution.

## 7. Future Prospects: Integrating Evo-Devo, Genomics, Transcriptomics and Genome Editing

The evolutionary origin of the ovule and seed is still quite enigmatic. As we have seen, there are a number of reasons for it. The fossil record is necessarily (and quite certainly forever) vastly incomplete and, as often in such cases, inconclusive. One can always hope that informative fossils will be identified in the future, but that is difficult to plan for, like all serendipity or sheer luck. That leaves us with the extant taxa.

Unfortunately, there is a great phylogenetic distance between extant seed plants and their extant sister group, the monilophytes (Figure 1). Roughly 400 million years of independent evolution in both the monilophyte and spermatophyte lineages have generated considerable genetic, morphological and physiological differences between these taxa. Structural and functional comparisons between monilophytes and spermatophytes, including homology assignments, are hence difficult, often vague, and error-prone.

Nevertheless, comparative studies of extant ferns and seed plants on ovule origin are not completely futile. However, such investigations were previously hampered by the fact that high-quality monilophyte genome sequences were not available, and that genetic manipulation of monilophyte species had not been established. This has now changed with the publication of whole-genome sequences of several fern species and protocols for the stable genetic transformation of ferns. A better understanding of ovule origin can now be obtained using new approaches harnessing extant species, despite their phylogenetic distance.

The fern species for which high-quality genome sequences have been published meanwhile are listed in Table 1.

These genome sequences now allow the efficient identification of homologues or even orthologues of seed plant genes in fern genomes. Arguably even more importantly, they enable for the first time the rigorous identification of seed plant genes that lack homologues or orthologues in monilophytes. For example, some studies about 30 years ago based on the screening of cDNA libraries strongly suggested that orthologues of floral organ identity genes are absent in the homosporous leptosporangiate fern *Ceratopteris richardii* [96], but one cannot exclude that some genes have been missed this way due to technical reasons. A more recent analysis of the whole *C. richardii* genome meanwhile strongly supported that hypothesis, however [32].

Another powerful method, especially if combined with genome analyses (genomics), is the comparison of transcriptomes (comparative transcriptomics). Especially valuable in this regard is the information on the spatio-temporal pattern of gene expression in different organs and tissues. This approach may work even beyond classical homology assignments, or may even indicate hidden or “deep” homologies and is thus especially valuable for a comparison between taxa as distantly related as monilophytes and spermatophytes.

A good case in point is a recent analysis aimed at investigating seed (and implicitly ovule) origin by quantifying gene network conservation between seed plant and monilophyte reproductive organs [97]. The authors used the fern *Ceratopteris richardii* as a model system and proxy for seedless reproduction in monilophytes. They generated a gene expression atlas of fern diploid and haploid reproductive organ development and tested for enrichment of respective genes in the angiosperm model plant *Arabidopsis thaliana*. The authors reported that ovule gene networks are enriched in genes regulating post-fertilization development of the fern archegonium (haploid egg chamber), a subset of which also revealed enrichment in sporophyll primordia. Based on these findings the authors suggested that the ovule originated from a sporangium-bearing axis based on the heterotopic expression of gene networks in the ancestral post-fertilization archegonium during development of the sporangium-bearing axis.

**Figure 4:**
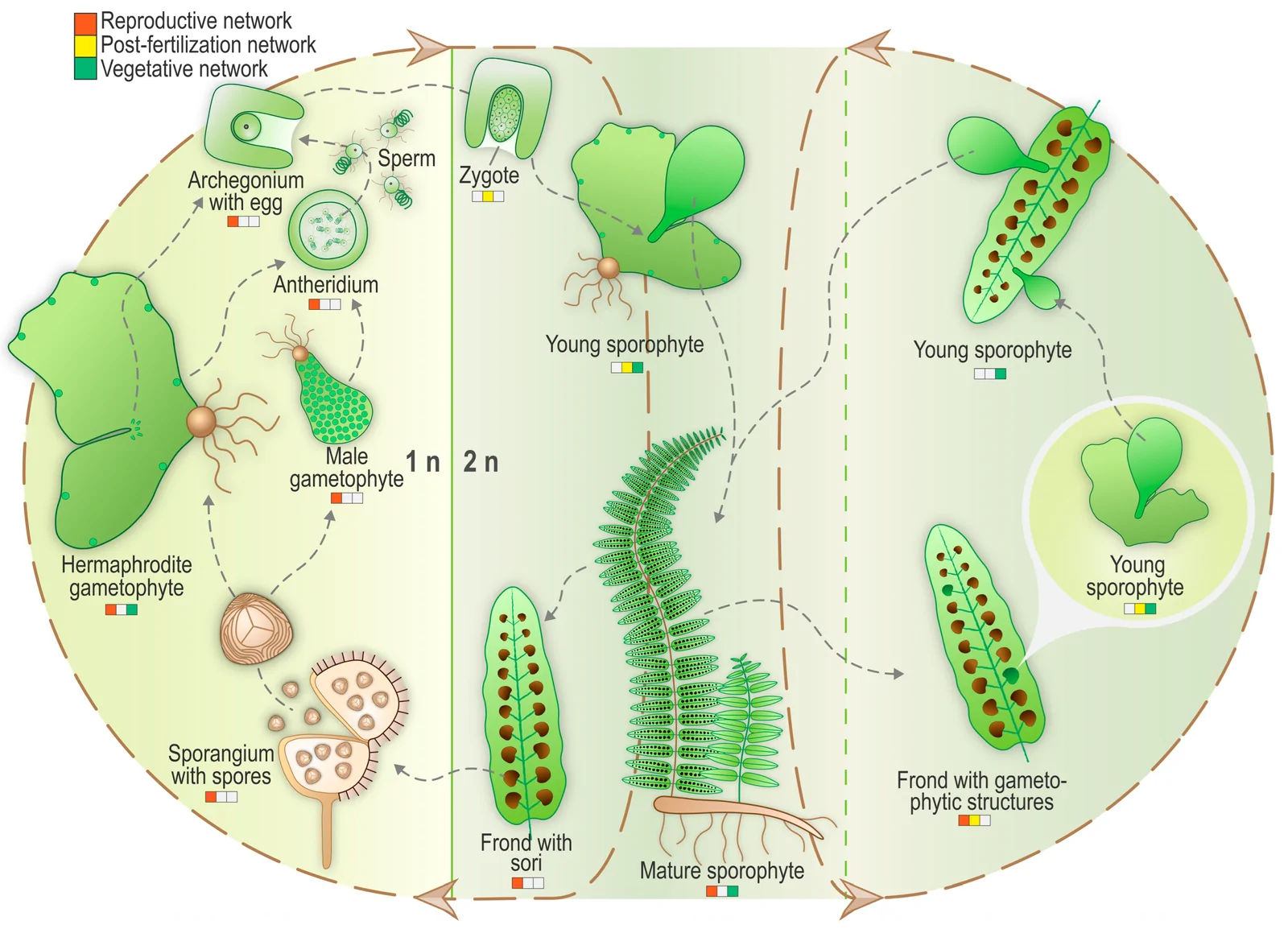
Generic life cycle of a homosporous fern and a hypothetical model of how changes in reproductive development might relate to the origin of the seed. The typical fern life cycle begins after fertilization, with the zygote located within the archegonium. The young sporophyte develops its first frond, followed by its first root. After a period of vegetative growth, it enters the reproductive phase and produces fronds bearing sori. The sporangia within the sori produce spores, which are released after maturation and germinate into gametophytes. The gametophytes then produce eggs within the archegonia and flagellated sperm within the antheridia. Preliminary transcriptomic observations may suggest possible similarities between regulatory networks associated with the post-reproductive archegonium and those associated with ovule development in seed plants [97]. This model explores the speculative possibility that some parts of the gene regulatory networks operating in fern archegonia may have been present in the most recent common ancestor of monilophytes and seed plants. It further assumes that this ancestor had a fern-like mode of reproduction. Under these assumptions, ferns could potentially be used as an experimental system to investigate and recreate innovations such as monomegaspory or endomegaspory. One possibility is that similar changes in an ancestral lineage could have created new developmental or evolutionary opportunities. It is also conceivable that surrounding sporangial tissues could subsequently have been co-opted into an integument-like structure. This model should be understood as a conceptual framework for generating testable hypotheses rather than as a reconstruction of established evolutionary events.

This unexpected scenario, whether correct or not, reveals the considerable heuristic value of comparing the developmental genetics of extant ferns and seed plants by modern methods despite their great phylogenetic distance. It immediately provokes the development of hypotheses that integrate the suggestions of the authors [97] into a more comprehensive model of the fern life cycle (Figure 4).

Another challenging but fruitful task might now be to reconcile and combine, or refute, other evo-devo scenarios that have been proposed for the origin of the ovule, such as that gene networks for sporangium development were co-opted for integument development [98] and that the integuments are the result of sporangia that became sterile and fused together around a fertile sporangium [99].

Gametogenesis in seed plants is extremely complex and not controlled solely by the gametophyte. It also depends on cross-talk between the sporophyte and the gametophyte. Furthermore, it has been shown that ovule identity depends on gene regulation asserted by the female gametophyte over the surrounding sporophytic tissue [100]. Also, there is gene regulation of the gametophyte mediated by proteins from the sporophyte. Any disruption of this delicate balance will prevent the ovule from fully developing [101].

Another point to consider is a rare phenomenon within ovules called apomixis, the asexual development of an embryo. There are three different types of apomixis: diplospory, apospory, and adventitious embryony. Diplospory and apospory involve the formation of megagametophytic structures without meiotic reduction and are therefore referred to as gametophytic apomixis. In contrast, adventitious embryony occurs in external tissue of mature ovules and is described as sporophytic apomixis [102,103]. The origin of apomixis is not clear but it seems to have arisen multiple times independently and there is evidence that it is connected to extreme environmental conditions [104,105]. Furthermore, the formation of sporophytic tissue on the gametophyte without prior fertilization can also be observed in bryophytes, lycophytes and monilophytes. Interestingly, while this phenomenon is naturally quite rare, it seems to be much more common in ferns [106]. Even less common, but observed in ferns is the spontaneous development of gametophytic structures on the sporophyte, a process called apospory. This seems to be connected to a combination of physical damage to sporophytic tissue and the permanent presence of water. The gametophytic structures resulting from apospory have the same ploidy level as the sporophyte [107,108]. Unfortunately, most of those observations are from a time before transcriptomic analyses became accessible. Therefore, the genetics behind apospory in ferns are unclear so far. The polyploidization resulting from such events can be reversed by re-diploidization through sexual reproduction. Reversing polyploidy often leads to diversification, potentially forming new genera [109]. It also drives hypo-, sub-, or neofunctionalization of genes such as those encoding transcription factors, leading to evolutionary innovations and a more complex morphology [110,111]. While class D genes are specific to ovule development, it has been experimentally shown that overexpression of the class D gene *AGL11* causes sporophytic apomixis in *A. thaliana* [112]. Also interesting are *BABY BOOM* (*BBM*) and *AINTEGUMENTA* (*ANT*), both members of the *APETALA2* family and important for ovule development. A study showed that overexpression of *BBM* causes the formation of somatic embryos in *A. thaliana* and *Brassica napus* [113]. On the other hand, overexpression of *ANT* caused an increase in the size of floral organs in *A. thaliana* [114]. This shows that despite being very closely related, *BBM* and *ANT* have different distinctive functions in angiosperms. Interestingly, the fern *C. richardii* has a homologue of *ANT* called *CrANT* but no homologue of *BBM*. Moreover, the heterologous expression of *BBM* from *B. napus* and the overexpression of *CrANT* in *C. richardii* both cause spontaneous formation of apogamous sporophytes. Evidently, there is a genetic conservation between somatic embryogenesis in angiosperms and apogamy in ferns [115]. This demonstrates a connection between apomixis and the evolutionary history of ovules and should be explored further in future research. Furthermore, it underlines the importance of finding homologues of ovule-related genes in non-seed plants and studying their functions to understand more about the origin of ovules and seeds. It cannot be determined based on fossil records alone if a plant was apomictic or sexual. Still, if the most recent common ancestor to seed plants and monilophytes had exhibited a stable aposporous program and undergone multiple rounds of re-diploidization, it may have expanded the evolutionary potential by allowing duplicated genes and regulatory networks to diverge and acquire new functions, thereby facilitating evolutionary innovations that may ultimately have contributed to the origin of the ovule.

Models on ovule origin based on comparative genomics and transcriptomics involving extant ferns and seed plants such as *Ceratopteris* and *Arabidopsis* will strongly benefit from the ability to test the function of candidate genes. Towards this goal, efficient genetic transformation protocols have meanwhile been established [116,117], and work to overexpress and knock out MADS-box genes and *LFY* genes possibly of interest for ovule origin in *C. richardii*, using for example the CRISPR-Cas9 system, is well underway [118,119,120].

With all these technical developments and conceptual prospects, the origin of the ovule (and seed) might be a bit more demystified in the not too distant future.

## Figures and Tables

**Table 1:** Leptosporangiate fern species for which high-quality sequenced genomes are available. Except the heterosporous *Azolla*, *Marsilea* and *Salvinia*, all other species are homosporous. The genomes of the heterosporous ferns *Salvinia molesta*, *Regnellidium diphyllum* and *Pilularia americana* are close to being published. Genomes of a few eusporangiate fern species are available as well, but not at high quality. Sequencing depth (Assembly level) provides an indication of the quality and completeness. Contigs are longer continuous sequences assembled from overlapping reads and usually do not contain gaps. Scaffolds consist of ordered and oriented contigs in larger blocks. They may contain gaps between contigs. A chromosome-level assembly means that scaffolds or contigs have been ordered, oriented and anchored into pseudochromosomes, often using Hi-C or Omni-C data. This does not necessarily mean that all gaps are closed. PacBio and Nanopore sequencing are used to generate long reads, which helps create long contigs and resolve repetitive regions. Illumina sequencing produces short, highly accurate reads. It is often used for polishing, quality control, SNP detection or resequencing many individuals. BAC stands for bacterial artificial chromosome. It involves cloning large DNA fragments into bacteria, followed by sequencing. Omni-C/Hi-C provides chromatin contact information and is used to order and orient contigs or scaffolds into chromosome-scale pseudochromosomes.

Species	Assembly Level	Sequencing Technology	Reference
*Adiantum capillus-veneris*	Chromosome	PacBio, Illumina	[85]
*Adiantum nelumboides*	Contig	PacBio, Illumina	[86]
*Alsophila spinulosa*	Chromosome	PacBio, Illumina	[87]
*Ceratopteris richardii*	Chromosome	PacBio, Illumina, BAC	[32]
*Cibotium barometz*	Chromosome	PacBio, Illumina	[88]
*Dipteris shenzhenensis*	Chromosome	PacBio, Illumina	[89]
*Lygodium microphyllum*	Chromosome	Nanopore, Illumina	[90]
*Polystichum acrostichoides*	Scaffold	Illumina	[91]
*Brainea insignis*	Chromosome	PacBio, Illumina	[92]
*Azolla caroliniana*	Chromosome	PacBio, Omnic-C	[93]
*Azolla filiculoides*	Contig	PacBio, Illumina	[94]
*Marsilea vestita*	Chromosome	Nanopore, Illumina	[95]
*Salvinia cucullata*	Contig	PacBio, Illumina	[94]

## Data Availability

No new data were created or analyzed in this study. Data sharing is not applicable to this article.
